# Afghanistan and the Global Heroin Trade

**DOI:** 10.1192/bjo.2022.205

**Published:** 2022-06-20

**Authors:** Eamonn Kinally

**Affiliations:** Royal Glamorgan Hospital, Llantrissant, United Kingdom

## Abstract

**Aims:**

In 2020, Afghanistan supplied around 85% of the world's heroin. The recent Taliban takeover and political upheaval seems highly likely to impact the supply chain, but how? This literature review aims to explore the background of heroin production and possible consequences of the recent conflict, both for suppliers and for end users.

**Methods:**

In addition to recent mainstream media news articles on the Afghanistan conflict, PubMed search terms “heroin adulteration” were used to find 202 results. Only results published from the year 2000 onwards were examined for relevance, leaving 160 results. These were reviewed for relevance and led to suggestions of similar PubMed articles to arrive at the final 23 sources used.

**Results:**

Studies of previous heroin shortages in Australia and the UK are discussed to gain insight into the potential effects of a future shortage. A reduction in heroin exports from Afghanistan would cut down the supply to most nations excluding North and South America. Sources of evidence for our current understanding of the supply chain are examined. Specific US and UK policy failure which led to the current situation is also provided for context.

Methods of production in Afghanistan and smuggling routes are also examined to help predict impending changes.

**Conclusion:**

Given the number of factors involved it is difficult to anticipate with much certainty how the Taliban takeover of Afghanistan will affect the global heroin trade, but based on the available literature it seems more likely that this will cause shortages rather than an increased supply.

Clinicians should be aware that in line with previous shortages, this may cause a shift towards increased rates of polysubstance use in regular heroin users. We may also see a rise in incidents of harm from heroin adulteration with substances other than the currently widespread paracetamol and caffeine.

